# Dietary Habits, Meal Timing, and Meal Frequency in Kuwaiti Adults: Analysis of the Kuwait National Nutrition Surveillance Data

**DOI:** 10.3390/nu15214537

**Published:** 2023-10-26

**Authors:** Fatema Alkhulaifi, Suad Al-Hooti, Sameer Al-Zenki, Husam AlOmirah, Charles Darkoh

**Affiliations:** 1 Department of Epidemiology, Human Genetics & Environmental Sciences, Center for Infectious Diseases, School of Public Health, University of Texas Health Science Center at Houston, Houston, TX 77030, USA; 2 Department of Epidemiology and Biostatistics, College of Public Health, Kuwait University, Khaldiya 12037, Kuwait; 3 Kuwait Institute of Scientific Research, Kuwait City 13109, Kuwait; suad.alhooti@gmail.com (S.A.-H.); szenki@kisr.edu.kw (S.A.-Z.); dralomirah@gmail.com (H.A.); 4 Microbiology and Infectious Diseases Program, Graduate School of Biomedical Sciences, University of Texas MD Anderson Cancer Center UTHealth, Houston, TX 77030, USA

**Keywords:** dietary habits, meal timing, meal frequency, late-night dinner, skipping meals, Kuwait

## Abstract

Dietary habits, including meal frequency, meal timing, and skipping meals, have been extensively studied due to their association with the development of noncommunicable diseases (NCDs). This study describes dietary habits, meal timing, frequency, skipping meals, and late-night eating in Kuwaiti adults. Kuwait National Nutrition Surveillance System data were utilized to reach the objectives of this study. The findings reveal that approximately 54% of the adults in Kuwait eat after 10 p.m., 29% skip breakfast, and 9.8% skip dinner. Furthermore, adults in Kuwait consume 4.4 meals per day on average. Women skip breakfast more often and have more extended night fasting than men (*p* < 0.001). Married adults skip breakfast and dinner less than unmarried adults (*p* < 0.001). In conclusion, this descriptive study provides valuable insights into the dietary habits of Kuwaiti adults, emphasizing the importance of further investigating the association between meal timing, meal frequency, and the prevalence of NCDs in Kuwait.

## 1. Introduction

The impact of dietary habits, such as meal frequency, meal timing, and skipping meals, has been studied extensively in the last decade because of their association with the development of noncommunicable diseases [1,2]. As of 2019, noncommunicable diseases have been responsible for 74% of deaths worldwide [3]. The role of macro- and micronutrients is essential in understanding the rise in the rates of noncommunicable diseases; nonetheless, epidemiological studies have demonstrated the significance of dietary habits on health [1,4]. 

The number and the timing of daily meals have been associated with diabetes [5], obesity [6], and metabolic syndrome [6,7]. Skipping meals has been associated with obesity [8], low intake of recommended nutrients [9,10], and an increase in odds of metabolic syndrome [11]. Moreover, night-time eating has been associated with a higher prevalence of metabolic syndrome [12] and an increased rate of obesity [13]. Dietary habits such as meal timing, frequency, skipping meals, and fasting impact metabolic outcomes [2]. 

Skipping breakfast and eating late-night dinners are associated with an increased risk of insulin resistance and cardiovascular disease [13,14,15,16]. This was demonstrated in a cross-over study in which individuals were given a diet with the same macronutrient composition but at different times on different dates, a dinner at 6 p.m. and a late dinner at 10 p.m. [17]. The study showed that eating dinner late is associated with glucose intolerance and reduces fatty acid oxidation and mobilization, especially in early sleepers. Another prospective cohort study assessed the association between meal frequency and type 2 diabetes among community-dwelling adults aged 45 years and older [5]. The study demonstrated that eating four meals a day, compared to eating three meals a day, was associated with a lower risk of type 2 diabetes, especially among normal-weight individuals (BMI < 25 kg/m^2^). The association of dietary habits and disease does not mean causation, especially in cross-sectional studies. Nonetheless, it is essential to assess associations and further investigate them with more extensive studies because if an association exists in different populations and circumstances, it could aid in understanding the cause.

Most published studies on meal timing and frequency were conducted in the United States, Europe, or Asia. As there is a need for more research in this area in the Middle East, Kuwait National Nutrition Surveillance System data provided an opportunity to describe meal timing and frequency in Kuwait. Seventy-two percent of deaths in Kuwait have been attributed to noncommunicable diseases [18], and the prevalence of metabolic syndrome is about 36% [19]. The Gulf region, including Kuwait, has a unique food culture where gatherings, weddings, and celebrations are usually in the evening, and food is abundant. Food in Kuwait is affordable compared to the rest of the world; thus, people tend to overeat. The hot, dry weather leads to adults and youth adopting a sedentary lifestyle [20]. Studies demonstrate that noncommunicable diseases and metabolic syndrome are associated with dietary habits. Little is known about meal timing and frequency in Kuwait, as only one study explored breakfast consumption in adolescents [21]. Thus, examining how dietary habits affect the Kuwaiti population is essential to better understanding the country’s high prevalence of chronic diseases. This paper aimed to describe dietary habits, including meal timing, meal frequency, skipping meals, and late-night eating, in Kuwaiti adults.

## 2. Materials and Methods

### 2.1. Study Sample

The study population comprises a nationally representative sample of the Kuwait National Nutrition Surveillance System (KNNSS), using the proportion-to-population-size method. At first, fifty-four localities were identified in Kuwait, considering the percentage of households in these localities. This study followed a stratified cluster sampling strategy to sample the households; the localities were divided into 82 clusters, and 20 households were selected from each cluster. A total of 1640 households and 7547 individuals were identified. Of these, 545 households and 1830 individuals agreed to participate in the survey (24.25% response rate). From each household, subjects over 20 years of age were randomly selected. Of the 1824 individuals that participated in the KNNSS, 757 adults were included in this study. In total, 722 individuals were not included because they were less than 20 years of age. Those younger than 20 years were assessed in the KNNSS differently, as adolescents and children. Another 345 individuals were excluded because they were taking diabetes, blood pressure, or cholesterol medication. 

### 2.2. Assessment of Dietary Data

The participants completed a 24 h dietary recall. Meal frequency was estimated based on the number of eating episodes. A meal is an eating episode in which an individual consumes any food or beverage of more than 1 Kcal [22,23]. Meal frequency is the number of meals an individual consumes within 24 h. Skipping breakfast was defined as not eating from 5 a.m. to 10 a.m. Skipping dinner was defined as not eating a meal from 6 p.m. to 10 p.m. Late-night eating was defined as eating between 10 p.m. and 3 a.m. Fasting was calculated by subtracting the time of the last meal from the time of the first meal. 

### 2.3. Covariate Assessment

Interviewer-administered questionnaires were used to collect data on demographic background, physical status, and lifestyle factors. Body measurements, such as body weight, height, and waist circumference, were obtained during household visits through standard protocol by trained personnel. A body composition analyzer (model TBF 310; Tanita) was used for body weight, a vertical stadiometer (model 214; Seca) was used for height, and a measuring tape was used for the waist. All biomarkers were measured by collecting blood samples during house visits after an overnight fast. Blood pressure measurements were taken while the person was seated with the feet flat on the floor using a sphygmomanometer or a professional electronic blood pressure monitor (Pro M, Spengler Electronic). Two measurements were taken for blood pressure (10 min apart), waist circumference, body weight, and height. Body mass index (BMI) was calculated as weight in kilograms divided by height in meters squared. Age was divided into two categories for adults: 20–49 years and 50 years and older. Smoking was defined as ever smoking a cigarette (yes/no). Physical activity was described as having an active (physically active) or a sedentary lifestyle (active/sedentary). Marriage was defined as those who are married and those who are not, including divorced and widowed (yes/no). Education was classified into six categories (less than middle school, middle school, high school, diploma, bachelor’s, and postgraduate). 

### 2.4. Statistical Analysis

The data were analyzed using SAS 9.4 software. Continuous variables were presented as median ± standard error of the mean, and all categorical variables were presented as frequency (*n*) and/or percentiles (%). The normality of the continuous variables was evaluated using the Shapiro–Wilk test. For meal frequency, individuals were divided into those who consume three or fewer, four or five meals, and six or more. An analysis of variance (ANOVA) was conducted between the four groups of meal frequency to determine if there was at least one different group in the mean or frequency. A *t*-test was conducted to identify if there was a difference in dietary habits between the two groups. A significance level of *p* < 0.05 was used for all statistical tests. 

## 3. Results

Table 1 shows the study participants’ general characteristics (*n* = 757). The study included only adults over 20 years, of which 623 (82.3%) were 20 to 49 years old and 134 (17.7%) were 50 years and older. There were 341 (45.05%) males and 416 (54.95%) females. The majority of the participants, 217 (28.67%), had a bachelor’s degree. Late-night eating was the most popular eating habit among participants in the study (53.9%), followed by skipping breakfast (29%) and skipping dinner (9.8%). The average duration of night fasting was 9.8 ± 3.3 h. The average BMI for males and females was 28.3 ± 5.9 kg/m^2^ and 29.9 ± 6.7 kg/m^2^, respectively. The mean waist circumference was 93.34 ± 16.3 cm. An estimated 33% (251) of the adults were smokers, while 41% (310) followed an active lifestyle. The mean blood pressure was 124.9/80.47 mmHg in this study. The mean HbA1c was 5.7 ± 0.88, while the mean triglycerides were 1.3 ± 0.74 mmol/L.

**Figure 1:**
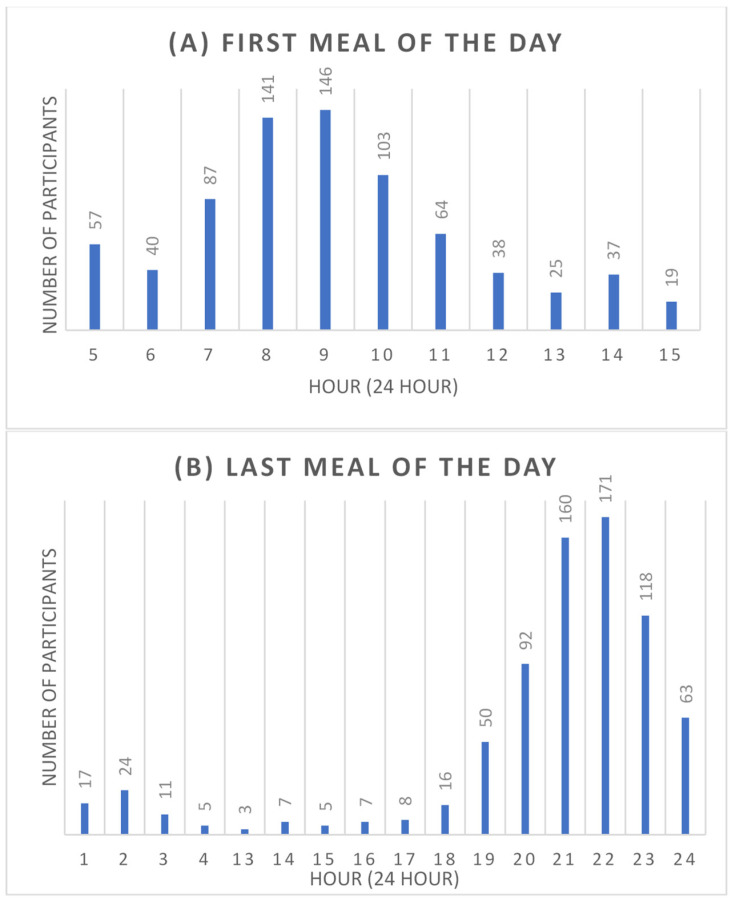
The number of participants consuming the first and last meals of the day by the hour. (**A**) A bar graph showing the number of participants who consumed the first meal of the day at each hour. (**B**) A bar graph showing the number of participants who consumed the last meal of the day at each hour.

The mean number of meals consumed per day was 4.4 meals (Table 2). The majority of the participants consumed four meals (29.9%) daily, followed by five meals (27.3%), three meals (20%), six meals (12.3%), and two meals (5%). Furthermore, most of the participants (19.3%) consumed their first meal of the day at either 9 a.m. (19.3%) or 8 a.m. (18.6%) (Figure 1). On the other hand, 22.6% of the participants consumed their last meal of the day at 10 p.m., followed by 21.1% at 9 p.m. (Figure 1). 

The general characteristics of the men and women in the study according to their meal frequency are shown in Table 3. Compared with individuals who ate three or fewer meals per day, individuals who ate six meals or more per day were more likely to be men, be married, consume meals after 10 p.m., have higher serum triglyceride, and fast for a shorter duration (*p* < 0.05). Furthermore, individuals who ate six or more meals per day were less likely to skip breakfast and dinner compared to individuals who ate three or fewer meals per day (*p* < 0.0001). Marital status, smoking, activity level, BMI, waist circumference, systolic and diastolic blood pressure, fasting glucose, HbA1, total serum cholesterol, and HDL cholesterol did not significantly differ based on meal frequency among the participants. 

An estimated 35.3% of the females and 23.5% of the males skipped breakfast (Table 4), which was significantly different (*p* = 0.0004). There was no difference in the proportion of males and females who skipped dinner. There was a significant difference (*p* = 0.0373) between the genders regarding late-night eating, which occurred in 58.1 % of the males and 50.5% of the females. The fasting duration was significantly different between the genders (*p* < 0.0001); men fasted at night on average for a period of 10.1 ± 2.8 h, and women fasted on average for 10.9 ± 2.9 h. 

Dietary habits differed based on the participants’ marital status (Table 4). A significant difference was found in the proportion of participants who skipped breakfast, as more unmarried participants skipped breakfast (36.5%) than married participants (27.6%). Similarly, a significantly higher proportion of unmarried participants (13.8%) skipped dinner than married participants (8.3%). Furthermore, unmarried participants fasted longer during the night than married participants, and the fasting durations were 10.9 and 10.4 h, respectively.

## 4. Discussion

This study demonstrated that 757 participants aged 20 and older consumed, on average, 4.4 meals daily within a range of one meal to ten meals. Most of the study participants consumed four meals (29.9%) or five meals (27.5%) daily. Moreover, 29% of the participants skipped breakfast, 53.9% ate meals later than 10 p.m., and only 9.8% skipped dinner. Dietary habits, including meal frequency, meal timing, skipping meals, and fasting, are as vital as nutrients in influencing health outcomes [1]. Thus, understanding and tracking the dietary habits of a community is essential to overcoming chronic health conditions [2].

The results of this study are unique to adults in Kuwait; yet it is important to see how they compare to other studies. The average number of meals consumed daily by the study participants (4.4 meals) is lower than the average of 5.7 meals and snacks consumed daily by US adults based on the NHANES survey (1971–2010) [24]. Although the NHANES survey calculated meals and snacks separately, they gave the average of meals and snacks (all eating events). In comparison, this study assessed all eating events as snacks or meals. Higher meal consumption has been inversely linked to abdominal obesity in Korean men [22], improving body composition, decreasing fat percentage, and improving cardiometabolic health [25]. Nonetheless, factors other than meal frequency, such as meal timing, play into the benefit of the meal to the individual. 

Skipping breakfast is associated with an increased risk of overweight and obesity [26,27]. A systematic review and meta-analysis reported an 11% increased risk ratio of overweight/obesity for individuals who skipped breakfast more than three days per week compared to those who skipped breakfast less than two days per week [27]. Another systematic review and meta-analysis found similar results [26]. In this study, 29% of the participants skipped breakfast or did not eat a meal until 11 a.m. The results are similar to a recent survey of breakfast consumption among adolescents in Kuwait, which illustrated that 21.7% of the adolescents never consumed breakfast or consumed it only once per week [21]. However, the habit of skipping breakfast differs in each country, as a study of Korean adults revealed that 41.2% skipped breakfast [28]. Another cohort study in Japan showed that 38.8% of the participants skipped dinner [29]. In contrast, a cross-sectional survey in Saudi Arabia revealed that 42% of medical students skipped breakfast [30]. A study of US adults shows that only 5.1% never consumed breakfast, while 10.9% rarely consumed breakfast [31]. 

Dinner is as essential as breakfast in determining health outcomes. A recent retrospective cohort revealed that skipping dinner significantly predicts weight gain and overweight/obesity in Japanese university students [32]. Eating a late dinner or bedtime snack and skipping dinner have been associated with an increased risk of obesity and overweight [32,33]. In this study, 9.8% skipped dinner, and 53.9% had a meal later than 10 p.m. The number of participants who ate late at night is comparable with other studies. A cohort study in Japan demonstrated that 57.1% had a late-night dinner [29]. In comparison, 49.7% of medical students in Saudi Arabia reported having a late-night dinner [30]. The proportion of the population who skipped dinner (9.8%) was low compared to those who skipped breakfast (29%). However, it was relatively high compared to a cohort study in Japan, where 2.4% of university students skipped dinner or ate it occasionally [32].

The frequency and timing of meals are associated with increased blood glucose levels and triglycerides [5,34]. This study demonstrated significant differences in serum triglycerides between individuals who ate different amounts of meals. As the frequency of meals increases, serum triglycerides increase. This finding was similar to that of an Australian study evaluating the association of meal frequency with the risk of metabolic syndrome, which illustrated that higher meal frequency was associated with elevated triglyceride levels among men [35]. 

There are some potential limitations to this study. First, this study extracted meal timing and frequency from a 24 h dietary recall. There were no specific questions on skipping meals and the number of snacks or meals consumed daily in the questionnaire administered during the study. Instead, data collected in 2010 in Kuwait’s National Nutrition Surveillance System were utilized as secondary data to calculate meal timing and frequency. A 24 h dietary recall captures one day’s diet, usually on a weekday; thus, it is not necessarily representative of an individual’s diet. A 3-day food record would be a more appropriate method for measuring a diet; however, describing the meal timing and meal frequency of adults in Kuwait using 24 h food recall provides valuable data not previously described in the literature.

Further, it is not uncommon to use 24 h food recall to assess meal timing and frequency, as more extensive studies have used 24 h recall for similar analyses [22,36]. Second, since data were collected from a 24 h food recall, all eating episodes were treated as meals, without differentiating between snacks and meals. This definition of a meal is used and supported by multiple studies to assess meal timing and frequency [22,23]. Comparing this study to studies that reported meals and snacks is challenging. Third, given that age was collected as a categorical variable in the Kuwait National Nutrition Surveillance System, it was only possible to assess age in two categories in adults: 20–49 years and more than 50 years. Fourth, a limitation is that the survey participation rate was approximately 24%. This may be due to the high participation involvement required to complete the survey. 

There are some strengths to this study. First, the study sample is a nationally representative sample of healthy medication-free adults 20 years and older recruited using a geographically representative method. The sample provides an excellent base to describe meal timing and frequency. Second, the study offers a prevalence of meal timing and frequency in Kuwait, where no data on meal timing and frequency existed before. Prevalences are valuable to compare data and to aid and guide future research.

## 5. Conclusions

Adults in Kuwait who are 20 years and older demonstrate dietary habits unique to Kuwait’s culture. Around 54% of the study participants eat a meal after 10 p.m., while only 29% skip breakfast and 9.8% skip dinner. The high proportions of late-night eaters and breakfast skippers could be attributed to cultural practices in Kuwait. However, it is crucial to investigate the implications of those dietary habits on health in future research. 

## Figures and Tables

**Table 1:** Characteristics of the participants included in the study.

Characteristic	N (%) or Mean ± SD
Age, *n* (%)	
20–49 years	623 (82.3)
50 years and older	134 (17.7)
Gender *n* (%)	
Male	341 (45.05)
Female	416 (54.95)
Education *n* (%)	
Less than middle school	39 (5.15)
Middle school	129 (17.04)
High school	155 (20.48)
Diploma	186 (24.57)
Bachelor’s degree	217 (28.67)
Postgraduate	31 (4.1)
Married, *n* (%)	
Yes	554 (73.2)
No	203 (26.8)
BMI, mean ± SD	29.15 ± 6.40
Male	28.3 ± 5.9
Female	29.9 ± 6.7
Waist circumference, mean ± SD	93.34 ± 16.30
Smoking (ever), *n* (%)	251 (33.29)
Skipping breakfast, *n* (%)	227 (29.0)
Skipping dinner, *n* (%)	74 (9.8)
Late-night eaters, *n* (%)	408 (53.9)
Fasting duration, mean ± SD	9.8 ± 3.3
Frequency of meals, mean ± SD	4.4 ± 1.3
Physical activity, *n* (%)	
Sedentary	446 (59)
Active	310 (41)
Glucose, mmol/L	5.6 ± 1.6
Triglycerides, mmol/L	1.3 ± 0.74
Blood pressure, mean ± SD	
Systolic	124.90 ± 17.13
Diastolic	80.47 ± 11.35

**Table 2:** Frequency of meals consumed by the participants.

Frequency of Meals	N (%), Mean ± SD
One meal	4 (0.5)
Two meals	38 (5.0)
Three meals	148 (19.6)
Four meals	226 (29.9)
Five meals	208 (27.5)
Six meals	93 (12.3)
Seven meals	28 (3.7)
Eight meals	9 (1.2)
Nine meals	1 (0.1)
Ten meals	2 (0.3)
Average number of meals	4.4 ± 1.04

**Table 3:** Characteristics of the study participants based on meal frequency.

	≤Three Meals	Four Meals	Five Meals	≥Six Meals	*p* ^a^
	N = 190	N = 226	N = 208	N = 133	
Age (20–49 years), %	81.1	84.5	80.8	82.7	0.727
Men, %	37.4 ^b^	42.5	47.6	56.4 ^c^	0.005 *
Married, %	66.3 ^b^	71.7	77.4	79.0 ^c^	0.023 *
College degree or higher, %	57.4	57.5	54.3	61.1	0.216
Ever smoked, %	29.0	30.9	36.7	38.4	0.183
Active, %	39.5	37.8	43.3	45.1	0.473
BMI (kg/m^2^), Mean ± SD	29.0 ± 6.8	29.2 ± 6.0	29.5 ± 6.5	28.8 ± 6.4	0.81
Body Fat %, Mean ± SD	24.9 ± 14.9	25.3 ± 12.2	26.3 ± 13.0	23.8 ± 14.7	0.415
Waist (cm), Mean ± SD	92.3 ± 16.7	92.4 ± 15.8	94.7 ± 16.8	94.4 ± 15.7	0.331
Systolic bp (mmHg), Mean ± SD	124.5 ± 17.7	124.5 ± 16.0	124.1 ± 17.5	127.3 ± 17.4	0.359
Diastolic bp (mmHg), Mean ± SD	79.5 ± 11.7	81.1 ± 11.2	80.3 ± 11.2	81.0 ± 11.4	0.554
Skipped breakfast, %	51.6 ^b^	32.7 ^c^	17.8 ^d^	13.5 ^d^	<0.0001
Skipped dinner, %	23.7 ^b^	6.6 ^c,d,e^	5.3 ^c,d,e^	2.26 ^c,d,e^	<0.0001
Late-night eater, %	44.2 ^b^	45.1 ^b^	60.0 ^c^	73.7 ^d^	<0.0001
Fasting, Mean ± SD	11.0 ± 4.1 ^b^	10.4 ± 3.1 ^b^	9.0 ± 2.7 ^c^	8.2 ± 2.3 ^d^	<0.0001
Glucose, Mean ± SD	5.4 ± 1.2	5.6 ± 1.6	5.6 ± 1.9	5.7 ± 1.8	0.303
HbA1C, Mean ± SD	5.6 ± 0.9	5.7 ± 0.8	5.7 ± 0.8	5.9 ± 1.1	0.114
Triglyceride, Mean ± SD	1.1 ± 0.6 ^b^	1.3 ± 0.7	1.4 ± 0.8 ^c^	1.3 ± 0.8	0.017 *
Total cholesterol, Mean ± SD	4.9 ± 1.0	5.1 ± 1.1	5.0 ± 1.0	5.1 ± 1.0	0.079
HDL cholesterol, Mean ± SD	1.3 ± 0.3	1.3 ± 0.3	1.2 ± 0.3	1.3 ± 0.3	0.082

^a^*p*-value for ANOVA F-statistics. *, ^b^, ^c^, ^d^, ^e^ groups are significantly different using Tukey’s post hoc test.

**Table 4:** Dietary habits based on gender and marriage status.

	Male N = 314	Female N = 416	*p*-Value ^a^	Married N = 554	Not Married N = 203	*p*-Value ^a^
Skipping breakfast, %	23.5	35.3	0.0004	27.6	36.5	0.0235
Skipping dinner, %	9.7	9.9	0.9345	8.3	13.8	0.0425
Late-night eater, %	58.1	50.5	0.0373	52.7	57.1	0.2775
Fasting, mean ± SD	10.1 ± 2.8	10.9 ± 2.9	<0.0001	10.4 ± 1.9	10.9 ± 3.3	0.0432

^a^ *p*-value for *t*-test.

## Data Availability

Data are unavailable due to privacy and ethical restrictions.
